# Retraction: Droplet fluid infusion into a dust layer in relation to self-cleaning

**DOI:** 10.1039/d6ra90015a

**Published:** 2026-02-09

**Authors:** Ghassan Hassan, Bekir Sami Yilbas, Hussain Al-Qahtani

**Affiliations:** a Mechanical Engineering Department, King Fahd University of Petroleum & Minerals Dhahran Saudi Arabia bsyilbas@kfupm.edu.sa; b Center of Research Excellence in Renewable Energy (CoRE-RE), King Fahd University of Petroleum and Minerals (KFUPM) Dhahran 31261 Saudi Arabia; c K.A.CARE Energy Research & Innovation Center Dhahran Saudi Arabia

## Abstract

Retraction of ‘Droplet fluid infusion into a dust layer in relation to self-cleaning’ by Ghassan Hassan *et al.*, *RSC Adv.*, 2020, **10**, 32034–32042, https://doi.org/10.1039/D0RA05700B.

The Royal Society of Chemistry hereby wholly retracts this *RSC Advances* article due to concerns with the reliability of the data.

Several concerns with the data have been identified within a group of articles by the same author group.

The authors have not been able to satisfactorily address these concerns.

Given the significance of these concerns, the Editor has lost confidence that the findings presented in this paper are reliable.

The authors were informed about the retraction of the article. Bekir Sami Yilbas has not agreed with the decision, the other authors have not responded.

Bekir Sami Yilbas states that authors disagree with the retraction and state that all figures were generated by the authors, and the reuse of one or two surface-characterization images neither constitutes duplicated data being presented as new nor compromises the validity of the findings, since the scientific discussion in each paper is supported by multiple figures.

Signed: Laura Fisher, Executive Editor, *RSC Advances*

Date: 30th January 2026

